# Impacts of Amplitude and Local Thermal Non-Equilibrium Design on Natural Convection within NanoflUid Superposed Wavy Porous Layers

**DOI:** 10.3390/nano11051277

**Published:** 2021-05-13

**Authors:** Ammar I. Alsabery, Tahar Tayebi, Ali S. Abosinnee, Zehba A. S. Raizah, Ali J. Chamkha, Ishak Hashim

**Affiliations:** 1 Refrigeration & Air-conditioning Technical Engineering Department, College of Technical Engineering, The Islamic University, Najaf 54001, Iraq; alsabery_a@iunajaf.edu.iq; 2 Faculty of Sciences and Technology, Mohamed El Bachir El Ibrahimi University, Bordj Bou Arreridj, El-Anasser 19098, Algeria; tahartayebi@gmail.com; 3 Energy Physics Laboratory, Department of Physics, Faculty of Science, Mentouri Brothers Constantine1 University, Constantine 25017, Algeria; 4 Computer Technical Engineering Department, College of Technical Engineering, The Islamic University, Najaf 54001, Iraq; abosinnee.ali@gmail.com; 5 Department of Mathematics, College of Science, King Khalid University, Abha 61421, Saudi Arabia; zhbarizah@hotmail.com; 6 Faculty of Engineering, Kuwait College of Science and Technology, Doha District 35001, Kuwait; achamkha@gmail.com; 7 Center of Excellence in Desalination Technology, King Abdulaziz University, P.O. Box 80200, Jeddah 21589, Saudi Arabia; 8 Department of Mathematical Sciences, Faculty of Science & Technology, Universiti Kebangsaan Malaysia, UKM Bangi 43600, Selangor, Malaysia

**Keywords:** natural convection, nanofluid-porous cavity, wavy solid wall, darcy-forchheimer model, local thermal non-equilibrium (LTNE)

## Abstract

A numerical study is presented for the thermo-free convection inside a cavity with vertical corrugated walls consisting of a solid part of fixed thickness, a part of porous media filled with a nanofluid, and a third part filled with a nanofluid. Alumina nanoparticle water-based nanofluid is used as a working fluid. The cavity’s wavy vertical surfaces are subjected to various temperature values, hot to the left and cold to the right. In order to generate a free-convective flow, the horizontal walls are kept adiabatic. For the porous medium, the Local Thermal Non-Equilibrium (LTNE) model is used. The method of solving the problem’s governing equations is the Galerkin weighted residual finite elements method. The results report the impact of the active parameters on the thermo-free convective flow and heat transfer features. The obtained results show that the high Darcy number and the porous media’s low modified thermal conductivity ratio have important roles for the local thermal non-equilibrium effects. The heat transfer rates through the nanofluid and solid phases are found to be better for high values of the undulation amplitude, the Darcy number, and the volume fraction of the nanofluid, while a limit in the increase of heat transfer rate through the solid phase with the modified thermal ratio is found, particularly for high values of porosity. Furthermore, as the porosity rises, the nanofluid and solid phases’ heat transfer rates decline for low Darcy numbers and increase for high Darcy numbers.

## 1. Introduction

Natural convection in a composite cavity, where part of it is porous and the other is fluid (natural convection in many layers of superimposed porous fluids), represents one of the most important topics that has received wide attention from researchers due to its multiple applications in engineering, such as insulation systems for fibrous and granular, packed bed solar energy storage, water conservers, and reactors cooling after the accident, and in geophysics, such as thermal circulation in lakes and contaminant transport in groundwater. Several researchers have dealt with this topic, as it was started by the study of Beavers and Joseph [1], where they presented the boundary conditions between the homogeneous fluid and the porous media in the simple situation. Convective heat transfer in porous beds saturated with a fluid was investigated for various thicknesses and permeabilities of bed [2]. Natural convection heat transfer in an enclosure, which is divided into two regions, one filled with a porous medium and the other with a fluid, was analyzed by Tong and Subramanian [3], for the aim of developing the characteristics of heat transfer for enclosures containing different quantities of porous material. Both studies of Poulikakos et al. [4] and Poulikakos [5] relied on measuring the flow of fluid floating on a porous bed heated from below at Rayleigh numbers above a critical value. The authors in Beckermann et al. [6] presented a two-dimensional study on natural convection, a rectangular fluid enclosure partially filled with different layers of porous material, vertical or horizontal. Numerical investigations were conducted for various enclosure aspect ratios, Rayleigh and Darcy numbers, and ratios and thicknesses of thermal conductivity of the porous region [7]. The authors in Hirata et al. [8] discussed the thermosolutal natural convection onset in horizontal superimposed fluid-porous layers. A numerical and analytical investigation was done for combined thermal and moisture conventions in an enclosure filled with a partially porous medium to enhance the moisture transport in the thermal energy storage unit [9]. The authors in Mikhailenko et al. [10] discussed the mechanism to address the effects of a uniform rotation and a porous layer in a local heat source electronic cabinet, where it studied the impacts of the Rayleigh, Taylor, and Darcy numbers and the porous layer thickness on hydro-thermodynamics. The authors in Saleh et al. [11] investigated the unstable convective flow in a vertical porous layer inside an enclosure due to a flexible fin.

To enhance the fluids’ thermal properties, researchers and engineers have used new kinds of particles with a nanometer size, which are named nanoparticles, in traditional fluids, which generated the term “nanofluid”. The applications of the heat transfer of nanofluids have been widely used for, e.g., cooling electronics, heating exchangers, car radiators, and machining [12]. The authors in Alsabery et al. [13] provided an explanation for the influence of the Darcy number, Rayleigh number, nanoparticle volume fraction, and power-law index on streamlines, isotherms, and the total heat transfer and on the thermal conductivity of the nanofluid and the porous medium. The numerical analysis of Al-Zamily [14] was implemented to investigate the fluid flow, entropy generation, and heat transfer inside an enclosure with an internal heat generation. The authors in Armaghani et al. [15] presented numerically the natural convection and generation of thermodynamic irreversibility in a cavity containing a partial porous layer filled with a Cu-water nanofluid. The authors in Miroshnichenko et al. [16] utilized a numerical simulation of porous layers’ effect on natural convection in an open cavity with a vertical hot wall and filled with a nanofluid.

Four main classifications in the modelling of transportation methods for porous materials include the Local Thermal Non-Equilibrium (LTNE), thermal dispersion, constant porosity, and variant porosity. LTNE assumptions can be used in modelling the heat exchange of convection in porous materials due to the different thermal conductivities in the fluid and porous material [12]. By applying an exact Chebyshev spectral element method, the natural convection in a porous cavity was improved using an LTNE model [17]. By considering LTNE effects, the authors in Ghalambaz et al. [18] addressed the natural convection in a cavity filled with a porous medium with the consideration of the thickness of the solid walls of the cavity. Taking into account the local thermal non-equilibrium model, natural convective circulation in a rotating porous cavity was investigated with a variable volumetric heat generation by [19].

The authors in Sivasankaran et al. [20] analyzed the convective heat and fluid flow of a nanofluid in an inclined cavity saturated with a heat-generating porous medium based on the LTNE model. The authors in Tahmasebi et al. [21] investigated the heat transfer of the natural convection in an enclosure filled with a nanofluid in three different layers of the fluid, the porous medium, and the solid, where the local thermal non-equilibrium model was used to model the porous layer. The studies of [22,23,24] investigated the natural convection of different nanofluids in each article within a porous cavity depending on a Local Thermal Non-Equilibrium Model (LTNEM). Natural, forced, and Marangoni convective flows in an open cavity partially saturated with a porous medium under the impacts of an inclined magnetic field were studied, where the LTNEM was used to represent the thermal field in the porous layer [25].

The study of natural convection in a wavy porous cavity is an interesting topic due to its wide range of usage in engineering, e.g., for the management of nuclear waste, the cooling of transpiration, building thermal insulators, geothermal power plants, and grain storage, and in geophysics, e.g., for modelling pollutant spreading (radionuclides), the movement of water in geothermal reservoirs, and petroleum reservoirs’ enhanced recovery [26]. Free convection in a cubical porous enclosure has been controlled by the wavy shape of the bottom wall and by inserting a conductive square cylinder inside the considered cavity [27]. The natural convection heat transfer inside a square wavy-walled enclosure filled with nanofluid and containing a hot inner corrugated cylinder was simulated by [28]. The enclosure was divided into two layers, one filled with Ag nanofluid and the other with porous media. The authors in Kadhim et al. [29] presented a parametric numerical analysis of the free convection in a porous enclosure with wavy walls filled by a hybrid nanofluid, at several inclination angles. The authors in Alsabery et al. [30] simulated the free convection heat transfer inside a porous cavity filled with water-based nanofluid with the consideration of the LTNE model. They assumed that there was an inner solid cylinder centered in the enclosure and that the bottom wall of the cavity was heated and wavy.

As acknowledged in an earlier literature survey, and to the best of the authors’ knowledge, and based on the need to consider the LTNE condition, there is no study dealing with the natural convection flow within nanofluid-superposed wavy porous layers with the local thermal non-equilibrium model. Therefore, this work proposes an understanding of the amplitude’s impacts and the local thermal non-equilibrium of a nanofluid-superposed wavy porous layers via the fluid flow and heat transfer features.

## 2. Mathematical Formulation

**Figure 1:**
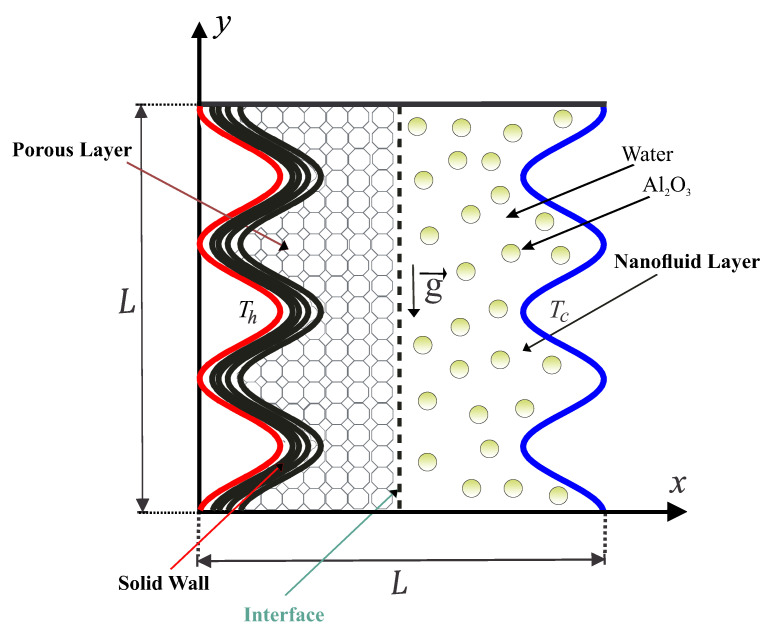
Schematic representation concerning the convection flow in the wavy-walled composite.

The two-dimensional natural convection state within the wavy-walled cavity with length *L* is explained in Figure 1. The analysed composite cavity is divided into three layers (portions). The first layer (left wavy portion) is solid as brickwork ($k_{w}=0.76$ tW/m.${}^{{}^{\circ}}$C), the second layer (middle portion) is loaded with a porous medium that is saturated with nanofluid, and the third layer (right wavy portion) is filled with a nanofluid. The wavy (vertical left) solid surface has a fixed hot temperature of $T_{h}$, while the vertical right wavy surface is fixed with a cold temperature of $T_{c}$. On the other hand, the horizontal top and bottom surfaces are preserved as adiabatic. The edges of the domain (except for the interface surface between the porous-nanofluid layer) are supposed to remain impermeable. The mixed liquid inside the composite cavity performs as a water-based nanofluid holding Al${}_{2}$O${}_{3}$ nanoparticles. The Forchheimer-Brinkman-extended Darcy approach and the Boussinesq approximation remain appropriate. In contrast, the nanofluid phase’s convection and the solid matrix are not in a local thermodynamic equilibrium condition. The set of porous media applied in the following output is glass balls ($k_{m}=1.05$ W/m.${}^{{}^{\circ}}$C). Considering the earlier specified hypotheses, the continuity, momentum, and energy equations concerning the Newtonian fluid, laminar, and steady-state flow are formulated as follows:

For the nanofluid layer,

(1) $$\begin{array}{ccc} & & \frac{\partial u_{nf}}{\partial x}+\frac{\partial v_{nf}}{\partial y}=0, \end{array}$$

(2) $$\begin{array}{ccc} & & u_{nf}\frac{\partial u_{nf}}{\partial x}+v_{nf}\frac{\partial u_{nf}}{\partial y}=-\frac{1}{\rho_{nf}}\frac{\partial p}{\partial x}+\frac{\mu_{nf}}{\rho_{nf}}\left(\frac{\partial^{2}u_{nf}}{\partial x^{2}}+\frac{\partial^{2}u_{nf}}{\partial y^{2}}\right), \end{array}$$

(3) $$\begin{array}{ccc} & & u_{nf}\frac{\partial v_{nf}}{\partial x}+v_{nf}\frac{\partial v_{nf}}{\partial y}=-\frac{1}{\rho_{nf}}\frac{\partial p}{\partial y}+\frac{\mu_{nf}}{\rho_{nf}}\left(\frac{\partial^{2}v_{nf}}{\partial x^{2}}+\frac{\partial^{2}v_{nf}}{\partial y^{2}}\right)+\beta_{nf}g\left(T_{h}-T_{c}\right), \end{array}$$

(4) $$\begin{array}{ccc} & & u_{nf}\frac{\partial T_{nf}}{\partial x}+v_{nf}\frac{\partial T_{nf}}{\partial y}=\frac{k_{nf}}{{\left(\rho C_{p}\right)}_{nf}}\left(\frac{\partial^{2}T_{nf}}{\partial x^{2}}+\frac{\partial^{2}T_{nf}}{\partial y^{2}}\right). \end{array}$$

For the porous layer,

(5) $$\begin{array}{c} \frac{\partial u_{m}}{\partial x}+\frac{\partial v_{m}}{\partial y}=0, \end{array}$$

(6) $$\begin{array}{c} \frac{\rho_{nf}}{\varepsilon^{2}}\left(u_{m}\frac{\partial u_{m}}{\partial x}+v_{m}\frac{\partial u_{m}}{\partial y}\right)=-\frac{\partial p}{\partial x}+\frac{\mu_{nf}}{\varepsilon }\;\left(\frac{\partial^{2}u_{m}}{\partial x^{2}}+\frac{\partial^{2}u_{m}}{\partial y^{2}}\right)\\ \;\;-\left(\frac{\mu_{nf}}{K}u_{m}-\frac{1.75}{\sqrt{150}\varepsilon^{3/2}}\frac{\rho_{nf}u_{m}\left|u\right|}{\sqrt{K}}\right), \end{array}$$

(7) $$\begin{array}{c} \frac{\rho_{nf}}{\varepsilon^{2}}\left(u_{m}\frac{\partial v_{m}}{\partial x}+v_{m}\frac{\partial v_{m}}{\partial y}\right)=-\frac{\partial p}{\partial y}+\frac{\mu_{nf}}{\varepsilon }\;\left(\frac{\partial^{2}v_{m}}{\partial x^{2}}+\frac{\partial^{2}v_{m}}{\partial y^{2}}\right)\\ \;\;-\left(\frac{\mu_{nf}}{K}v_{m}-\frac{1.75}{\sqrt{150}\varepsilon^{3/2}}\frac{\rho_{nf}v_{m}\left|u\right|}{\sqrt{K}}\right)+{\left(\rho \beta \right)}_{nf}g\left(T_{h}-T_{c}\right), \end{array}$$

(8) $$\begin{array}{ccc} & & u_{m}\frac{\partial T_{m}}{\partial x}+v_{m}\frac{\partial T_{m}}{\partial y}=\frac{\varepsilon k_{nf}}{{\left(\rho C_{p}\right)}_{nf}}\left(\frac{\partial^{2}T_{m}}{\partial x^{2}}+\frac{\partial^{2}T_{m}}{\partial y^{2}}\right)+\frac{h\left(T_{s}-T_{m}\right)}{{\left(\rho C_{p}\right)}_{nf}}, \end{array}$$

(9) $$\begin{array}{ccc} & & 0=\left(1-\varepsilon \right)k_{s}\left(\frac{\partial^{2}T_{s}}{\partial x^{2}}+\frac{\partial^{2}T_{s}}{\partial y^{2}}\right)+h\left(T_{m}-T_{s}\right). \end{array}$$

The energy equation of the wavy left solid surface is

(10) $$\frac{\partial^{2}T_{w}}{\partial x^{2}}+\frac{\partial^{2}T_{w}}{\partial y^{2}}=0.$$

The subscripts nf, *m*, *s*, and *w* correspond to the nanofluid layer, porous layer (nanofluid phase), porous layer (solid phase), and solid wavy surface, respectively. *x* and *y* are the fluid velocity elements, $\left|u\right|=\sqrt{u^{2}+v^{2}}$ denotes the Darcy velocity, g displays the acceleration due to gravity, ε signifies the porosity of the medium, and *K* is the permeability of the porous medium which is determined as

(11) $$K=\frac{\varepsilon^{3}d_{m}^{2}}{150{\left(1-\varepsilon \right)}^{2}}.$$

Here, $d_{m}$ represents the average particle size of the porous bed.

The thermophysical characteristics regarding the adopted nanofluid for the 33 nm particle-size are given by [31]:

(12) $$\begin{array}{ccc} & & {\left(\rho C_{p}\right)}_{nf}=\left(1-\phi \right){\left(\rho C_{p}\right)}_{f}+\phi {\left(\rho C_{p}\right)}_{p}, \end{array}$$

(13) $$\begin{array}{ccc} & & \rho_{nf}=\left(1-\phi \right)\rho_{f}+\phi \rho_{p}, \end{array}$$

(14) $$\begin{array}{ccc} & & {\left(\rho \beta \right)}_{nf}=\left(1-\phi \right){\left(\rho \beta \right)}_{f}+\phi {\left(\rho \beta \right)}_{p}, \end{array}$$

(15) $$\begin{array}{ccc} & & \frac{\mu_{nf}}{\mu_{f}}=\frac{1}{1-34.87{\left(\frac{d_{p}}{d_{f}}\right)}^{-0.3}\phi^{1.03}}, \end{array}$$

(16) knfkf=1+4.4ReB0.4Pr0.66TTfr10kpkf0.03ϕ0.66.

where ReB is defined as

(17) ReB=ρfuBdpμf,uB=2kbTπμfdp2.

The molecular diameter of water ($d_{f}$) is given as [31]

(18) $$d_{f}=0.1{\left[\frac{6M}{N^{*}\pi \rho_{f}}\right]}^{\frac{1}{3}}.$$

Now, we present the employed non-dimensional variables:

(19) $$\begin{array}{ccc} & & \left(X,Y\right)=\frac{\left(x,y\right)}{L},\;\;U_{nf,m}=\frac{u_{nf,m}L}{\alpha_{f}},\;\;V_{nf,m}=\frac{v_{nf,m}L}{\alpha_{f}},\;\;\theta_{nf}=\frac{T_{nf}-T_{c}}{T_{h}-T_{c}},\\ & & \theta_{m}=\frac{T_{m}-T_{c}}{T_{h}-T_{c}},\;\;P=\frac{pL^{2}}{\rho_{f}\alpha_{f}^{2}},\;\;k_{eff}=\varepsilon k_{nf}+\left(1-\varepsilon \right)k_{m},\;\;C_{F}=\frac{1.75}{\sqrt{150}}. \end{array}$$

The set scheme leads to the following dimensionless governing equations:

In the nanofluid layer,

(20) $$\begin{array}{ccc} & & \frac{\partial U_{nf}}{\partial X}+\frac{\partial V_{nf}}{\partial Y}=0, \end{array}$$

(21) $$\begin{array}{c} U_{nf}\frac{\partial U_{nf}}{\partial X}+V_{nf}\frac{\partial U_{nf}}{\partial Y}=-\frac{\partial P}{\partial X}+\mathrm{Pr}\frac{\rho_{f}}{\rho_{nf}}\frac{\mu_{nf}}{\mu_{f}}\left(\frac{\partial^{2}U_{nf}}{\partial x^{2}}+\frac{\partial^{2}U_{nf}}{\partial Y^{2}}\right), \end{array}$$

(22) $$\begin{array}{c} U_{nf}\frac{\partial V_{nf}}{\partial X}+V_{nf}\frac{\partial V_{nf}}{\partial Y}=-\frac{\partial P}{\partial Y}+\mathrm{Pr}\frac{\rho_{f}}{\rho_{nf}}\frac{\mu_{nf}}{\mu_{f}}\left(\frac{\partial^{2}V_{nf}}{\partial X^{2}}+\frac{\partial^{2}V_{nf}}{\partial Y^{2}}\right)\\ \;\;+\frac{{\left(\rho \beta \right)}_{nf}}{\rho_{nf}\beta_{f}}Ra\;\mathrm{Pr}\;\theta_{nf}, \end{array}$$

(23) $$\begin{array}{c} U_{nf}\frac{\partial \theta_{nf}}{\partial X}+V_{nf}\frac{\partial \theta_{nf}}{\partial Y}=\frac{{\left(\rho C_{p}\right)}_{f}}{{\left(\rho C_{p}\right)}_{nf}}\frac{k_{nf}}{k_{f}}\left(\frac{\partial^{2}\theta_{nf}}{\partial X^{2}}+\frac{\partial^{2}\theta_{nf}}{\partial Y^{2}}\right). \end{array}$$

In the porous layer,

(24) $$\begin{array}{ccc} & & \frac{\partial U_{m}}{\partial X}+\frac{\partial V_{m}}{\partial Y}=0, \end{array}$$

(25) $$\begin{array}{c} \frac{1}{\varepsilon^{2}}\left(U_{m}\frac{\partial U_{m}}{\partial X}+V_{m}\frac{\partial U_{m}}{\partial Y}\right)=-\frac{\partial P}{\partial X}+\frac{\rho_{f}}{\rho_{nf}}\frac{\mu_{nf}}{\mu_{f}}\frac{\mathrm{Pr}}{\varepsilon }\left(\frac{\partial^{2}U_{m}}{\partial X^{2}}+\frac{\partial^{2}U_{m}}{\partial Y^{2}}\right)\\ \;\;-\frac{\rho_{f}}{\rho_{nf}}\frac{\mu_{nf}}{\mu_{f}}\frac{\mathrm{Pr}}{Da}U_{m}-\frac{C_{F}\sqrt{U_{m}^{2}+V_{m}^{2}}}{\sqrt{Da}}\frac{U_{m}}{\varepsilon^{3/2}}, \end{array}$$

(26) $$\begin{array}{c} \frac{1}{\varepsilon^{2}}\left(U_{m}\frac{\partial V_{m}}{\partial X}+V_{m}\frac{\partial V_{m}}{\partial Y}\right)=-\frac{\partial P}{\partial Y}+\frac{\rho_{f}}{\rho_{nf}}\frac{\mu_{nf}}{\mu_{f}}\frac{\mathrm{Pr}}{\varepsilon }\left(\frac{\partial^{2}V_{m}}{\partial X^{2}}+\frac{\partial^{2}V_{m}}{\partial Y^{2}}\right)\\ \;\;-\frac{\rho_{f}}{\rho_{nf}}\frac{\mu_{nf}}{\mu_{f}}\frac{\mathrm{Pr}}{Da}V_{m}-\frac{C_{F}\sqrt{U_{m}^{2}+V_{m}^{2}}}{\sqrt{Da}}\frac{V_{m}}{\varepsilon^{3/2}}+\frac{{\left(\rho \beta \right)}_{nf}}{\rho_{nf}\beta_{f}}Ra\;\mathrm{Pr}\;\theta_{m}, \end{array}$$

(27) $$\begin{array}{c} \frac{1}{\varepsilon }\left(U_{m}\frac{\partial \theta_{m}}{\partial X}+V_{m}\frac{\partial \theta_{m}}{\partial Y}\right)=\frac{k_{eff}}{k_{f}}\frac{{\left(\rho C_{p}\right)}_{f}}{{\left(\rho C_{p}\right)}_{nf}}\left(\frac{\partial^{2}\theta_{m}}{\partial X^{2}}+\frac{\partial^{2}\theta_{m}}{\partial Y^{2}}\right)\\ \;\;+\frac{{\left(\rho C_{p}\right)}_{f}}{{\left(\rho C_{p}\right)}_{nf}}H\left(\theta_{s}-\theta_{m}\right), \end{array}$$

(28) $$\begin{array}{ccc} & & 0=\frac{\partial^{2}\theta_{s}}{\partial X^{2}}+\frac{\partial^{2}\theta_{s}}{\partial Y^{2}}+\gamma H\left(\theta_{m}-\theta_{s}\right). \end{array}$$

In the wavy solid wall,

(29) $$\frac{\partial^{2}\theta_{w}}{\partial X^{2}}+\frac{\partial^{2}\theta_{w}}{\partial Y^{2}}=0.$$

The dimensionless boundary conditions of Equations (20)–(28) are

(30) $$\begin{array}{l} \mathrm{On}\mathrm{the}\mathrm{left}\mathrm{solid}\mathrm{hot}\mathrm{wavy}\mathrm{surface},\\ U=V=0,\;\;\theta_{w}=1,\;\;A\left(1-\cos\left(2N\pi Y\right)\right),\;\;0\leq Y\leq 1, \end{array}$$

(31) $$\begin{array}{l} \mathrm{On}\mathrm{the}\mathrm{bottom}\mathrm{adiabatic}\mathrm{horizontal}\mathrm{surface},\\ U=V=0,\;\;\frac{\partial \theta_{\left(nf,m,s,w\right)}}{\partial Y}=0,\;\;0\leq X\leq 1,\;\;Y=0, \end{array}$$

(32) $$\begin{array}{l} \mathrm{On}\mathrm{the}\mathrm{right}\mathrm{cold}\mathrm{wavy}\mathrm{surface},\\ U=V=0,\;\;\theta_{nf}=0,\;\;1-A\left(1-\cos\left(2N\pi Y\right)\right),\;\;0\leq Y\leq 1, \end{array}$$

(33) $$\begin{array}{l} \mathrm{On}\mathrm{the}\mathrm{top}\mathrm{adiabatic}\mathrm{horizontal}\mathrm{surface},\\ U=V=0,\;\;\frac{\partial \theta_{\left(nf,m,s,w\right)}}{\partial Y}=0,\;\;0\leq X\leq 1,\;\;Y=1, \end{array}$$

The dimensionless boundary forms toward the interface between the nanofluid and the porous layers will be obtained from (1) the continuity of tangential and normal velocities, (2) shear and normal stresses, and (3) the temperature and the heat flux crossing the central interface and allowing an identical dynamic viscosity ($\mu_{nf}=\mu_{m}$) into both layers. Therefore, the interface dimensionless boundary conditions can be addressed as the following:

(34) $$\begin{array}{ccc} \theta_{nf}{|}_{Y=D^{+}} & = & \theta_{m}{|}_{Y=D^{-}}, \end{array}$$

(35) $$\begin{array}{ccc} \frac{\partial \theta_{nf}}{\partial Y}{|}_{Y=D^{+}} & = & \frac{k_{eff}}{k_{nf}}\frac{\partial \theta_{m}}{\partial Y}{|}_{Y=D^{-}}, \end{array}$$

(36) $$\begin{array}{ccc} U_{nf}{|}_{Y=D^{+}} & = & U_{m}{|}_{Y=D^{-}}, \end{array}$$

(37) $$\begin{array}{ccc} V_{nf}{|}_{Y=D^{+}} & = & V_{m}{|}_{Y=D^{-}}, \end{array}$$

Here, *D* denotes the nanofluid layer’s thickness, and the subscripts + and − indicate that the corresponding measures are estimated while addressing the interface of the nanofluid and the porous layers, respectively. $Ra=\frac{g\beta_{f}\left(T_{h}-T_{c}\right)L^{3}}{\nu_{f}\alpha_{f}}$ and $\mathrm{Pr}=\frac{\nu_{f}}{\alpha_{f}}$ signify the Rayleigh number and the Prandtl number related to the used base liquid.

The local Nusselt numbers ($Nu_{nf}$ and $Nu_{s}$) at the wavy vertical (left) surface for the nanofluid and the solid phases, respectively, are written as follows:

(38) $$Nu_{nf}=\frac{k_{eff}}{k_{f}}{\left(\frac{\partial \theta_{nf}}{\partial n}\right)}_{n},$$

(39) $$Nu_{s}=\frac{k_{s}}{k_{f}}{\left(\frac{\partial \theta_{s}}{\partial n}\right)}_{n}.$$

Here, *n* denotes the entire length of the curved heat source.

Lastly, the average Nusselt numbers at the wavy vertical surface within the nanofluid and solid phases are addressed by the following:

(40) $$\begin{array}{c} {\bar{Nu}}_{nf}=\int_{0}^{n}Nu_{nf}\;dn, \end{array}$$

(41) $$\begin{array}{c} {\bar{Nu}}_{s}=\int_{0}^{n}Nu_{s}\;dn. \end{array}$$

## 3. Numerical Method and Validation

**Figure 2:**
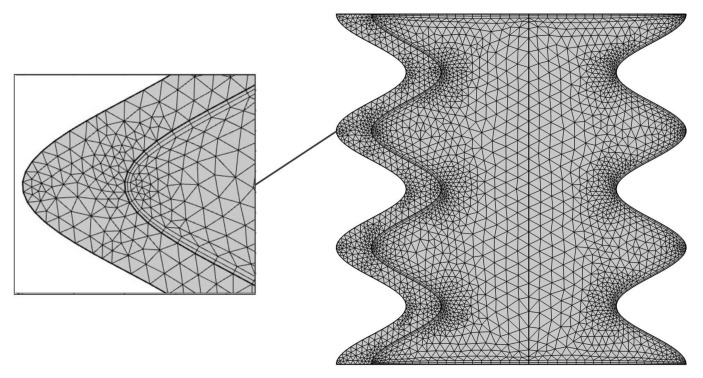
Framework configuration of the FEM for the grid dimension of 5464 components.

The governing dimensionless equations Equations (20)–(28) ruled with the boundary conditions Equations (30)–(37) are solved by the Galerkin weighted residual finite element technique. The computational region is discretised into small triangular portions as shown in Figure 2.

These small triangular Lagrange components with various forms are applied to each flow variable within the computational region. Residuals for each conservation equation is accomplished through substituting the approximations within the governing equations. The Newton-Raphson iteration algorithm is adopted for clarifying the nonlinear expressions into the momentum equations. The convergence from the current numerical solution is considered, while the corresponding error of each of the variables satisfies the following convergence criteria:

$$\begin{array}{c} \left|\frac{\Gamma^{i+1}-\Gamma^{i}}{\Gamma^{i+1}}\right|\leq 10^{-6}. \end{array}$$

To assure the confidence of the existing numerical solution at the grid size of the numerical region, we have adopted various grid dimensions for calculating the minimum strength of the flow circulation ($\Psi_{\min}$), the average Nusselt number of the nanofluid phase (${\bar{Nu}}_{nf}$), and the average Nusselt number of the solid phase (${\bar{Nu}}_{s}$) for the case of $Ra=10^{6}$, $Da=10^{-3}$, ϕ=0.02, N=3, γ=10, H=10, ε=0.5, and A=0.1. The outcomes are displayed in Table 1, which designates insignificant variations for the G6 grids and higher. Hence, concerning all calculations into this numerical work, the G6 uniform grid is applied.

**Figure 3:**
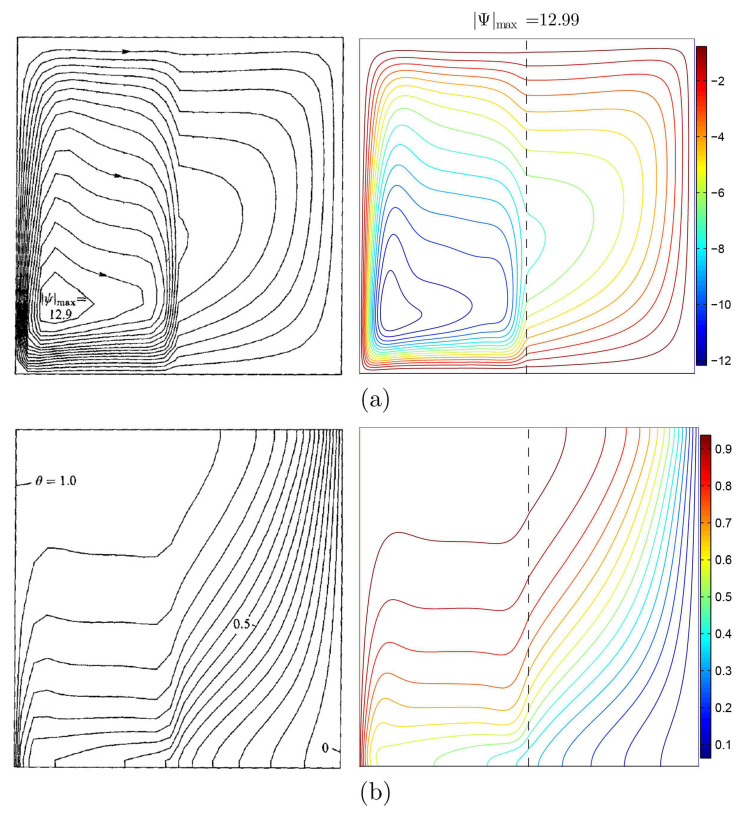
(**a**) Streamlines of Beckermann et al. [6] (**left**) and the present study (**right**); (**b**) isotherms for $Ra=3.70\times 10^{6}$, $Da=1.370\times 10^{-5}$, ε=0.9, D=0.5N=0, $\frac{k_{eff}}{k_{f}}=1.362$, and Pr=6.44.

**Figure 4:**
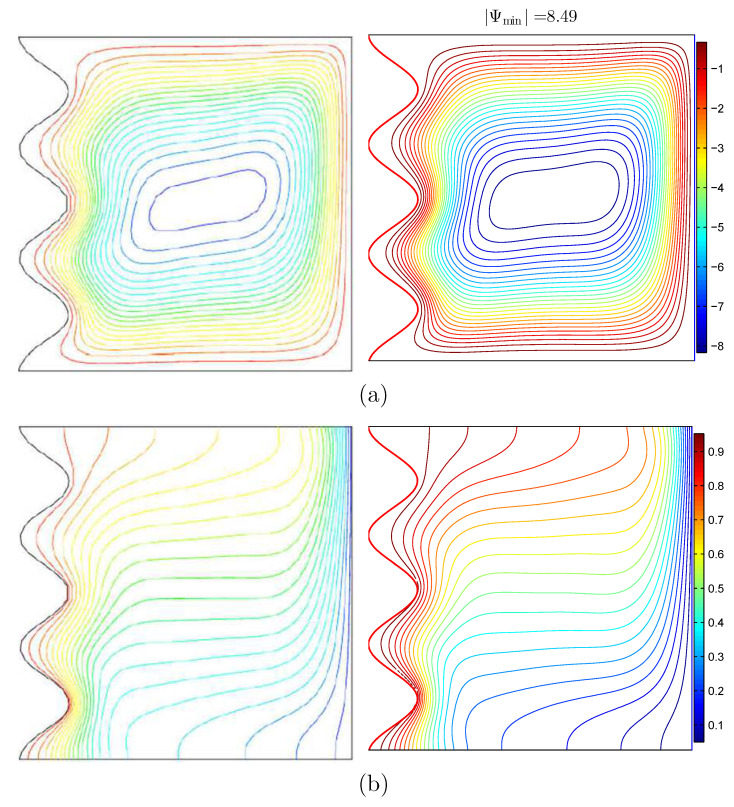
(**a**) Streamlines of (**left**) Khanafer et al. [32] and (**right**) the present study; (**b**) isotherms of (**left**) Khanafer et al. [32] and (**right**) the present study for $Ra=10^{5}$, $Da=10^{-2}$, ε=0.9, N=3, D=0, and Pr=1.

Concerning the validation for the current numerical data, the outcomes are examined with earlier published experimental results reported by Beckermann et al. [6] for natural convection within a square cavity including fluid and porous layers, as performed in Figure 3. Besides that, a comparison is obtained for the resulting patterns and the one implemented by Khanafer et al. [32] for the case of natural convection heat transfer in a wavy non-Darcian porous cavity, as displayed in Figure 4. According to the above-achieved comparisons, the numerical outcomes of the existing numerical code are significant to a great degree of reliability.

## 4. Results and Discussion

The outcomes described by streamlines, isotherms, and isentropic distributions are addressed within this section. We have modified the following four parameters: the Darcy number ($10^{-6}\leq Da\leq 10^{-2}$), the nanoparticle volume fraction (0≤ϕ≤0.04), the modified conductivity ratio (0.1≤γ≤1000), the amplitude (0≤A≤0.2), and the porosity of the medium (0.2≤ε≤0.8). The values of the Rayleigh number, the number of undulations, the coefficient of inter-phase heat transfer, the thickness of the wavy solid wall, and the Prandtl number are fixed at $Ra=10^{6}$, N=3, H=10, W=0.2, and Pr=4.623, respectively. Table 2 displays the thermos-physical properties of the base fluid (water) and the solid Al${}_{2}$O${}_{3}$ phases at T=310 K.

**Figure 5:**
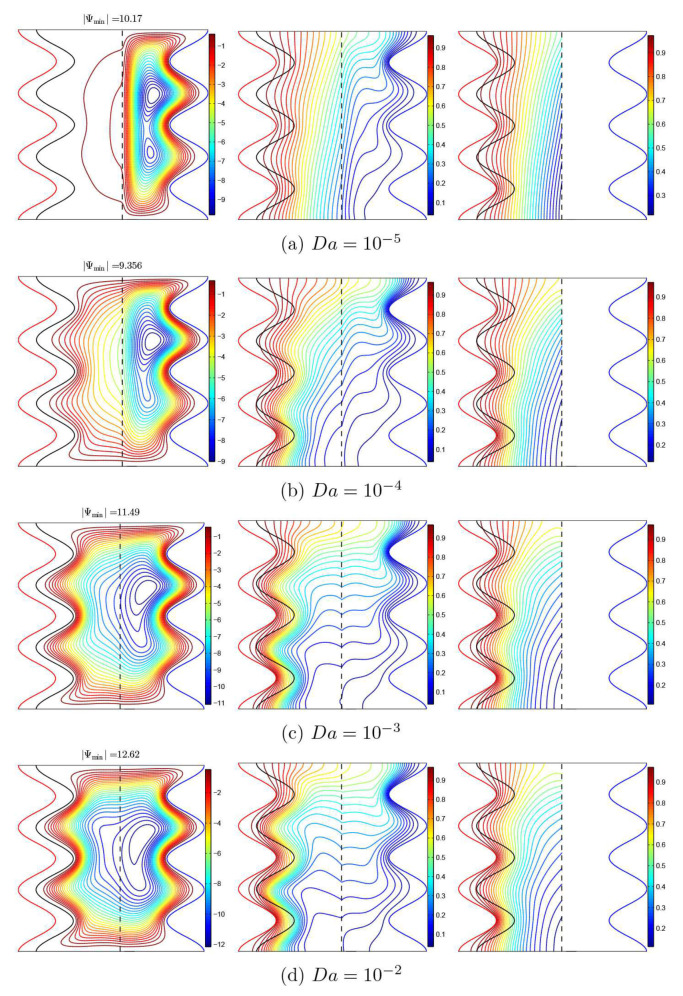
Streamlines (**left**), isotherms of the nanofluid phase (**middle**), and isotherms of the solid phase (**right**) with various Darcy numbers (Da); ϕ=0.02, γ=10, A=0.1, and ε=0.5.

Figure 5 displays from left to right, respectively, streamlines, isotherms of the nanofluid phase, and isotherms of the solid phase for various Darcy numbers (Da) when ϕ=0.02, γ=10, A=0.1, and ε=0.5. For a low Darcy number, the isotherms are virtually vertical in the porous layer and correspond to the solid phase isotherms, indicating that the heat transfer occurs essentially by the conduction mode because the porous medium becomes less permeable. The porous matrix causes the flow to cease in the porous layer; as a result, the flow is closed or entirely limited to the nanofluid layer and is not able to permeate into the porous medium. The heat transfer in the nanofluid layer is mainly convective, as is shown from the isotherms and the streamlines. When the Darcy number increases, the porous layer provides less resistance to the nanofluid flow, the natural convection increases, and the mechanism of heat transfer shifts from the conduction mechanism at a small Darcy number into the convection mechanism at a high Darcy number in the porous layer as well as in the entire enclosure. A high thermal boundary layer is present at the porous-layer/conducting solid-wall interface. Moreover, by comparing the isothermal lines in the solid part and the nanofluid phase in the porous layer, it is clear that, when the heat transport mode is mainly governed by conduction at low Darcy number values, the Local Thermal-Equilibrium (LTE) state is feasible because the isotherms are conformal (identical and similar). Meanwhile, the effect of the Local Thermal Non-Equilibrium (LTNE) is important at high Darcy numbers since there is a marked difference in the temperature distribution between the solid phase and the nanofluid phase in the porous layer.

**Figure 6:**
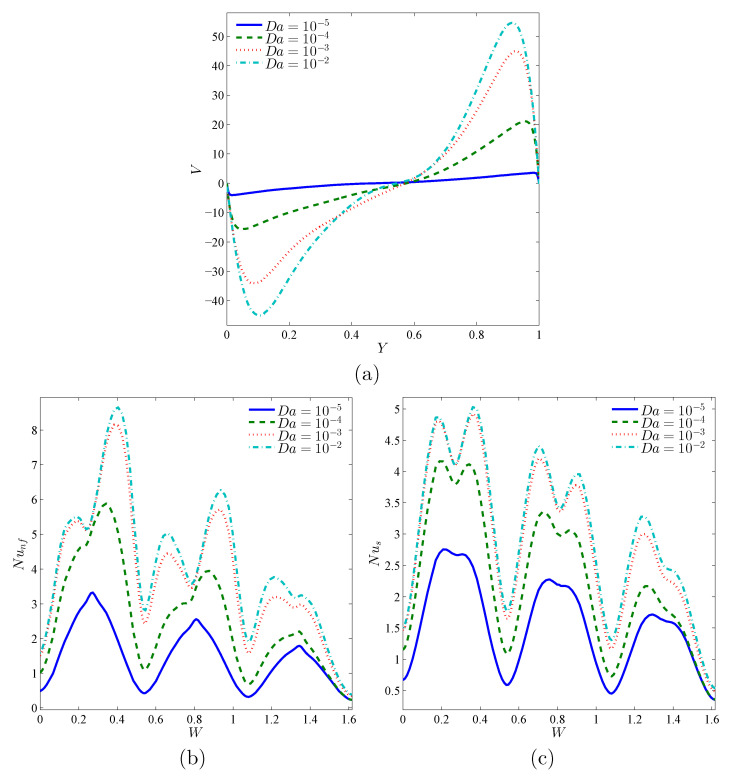
Local velocity (**a**), the local Nusselt number of the nanofluid phase (**b**), and the local Nusselt number of the solid phase (**c**) with the vertical line (*Y*) for X=0.5 for different Da values; ϕ=0.02, γ=10, A=0.1, and ε=0.5.

To frame the effect of the porous medium permeability, we present in Figure 6 the numerical results given by the profiles of the local velocity with the vertical line at X=0.5 (a), the local Nusselt number of the nanofluid phase (b), and the local Nusselt number of the solid phase (c) for different Da for the case of ϕ=0.02, γ=10, A=0.1, and ε=0.5. The velocity profiles indicate the rotational aspect of the nanofluid in the cavity. The nanofluids’ circulation rate increases with increasing values of the Darcy number. It is also evident from this figure that the local Nusselt numbers, for both the nanofluid phase and the solid phase, form peaks, and each peak corresponds to a convex boundary of the undulating hot wall. In comparison, the values of the Nusselt number improve by incrementing the Darcy number, and this improvement is more progressive for medium Darcy number levels. It is also worth noting that the heat transfer rate on the lower portion of the wavy wall is bigger than on the top. This is because these regions represent the contact areas for the cold nanofluid returning from the opposite cold wall, so a high temperature difference exists there, which causes a high heat transfer rate.

**Figure 7:**
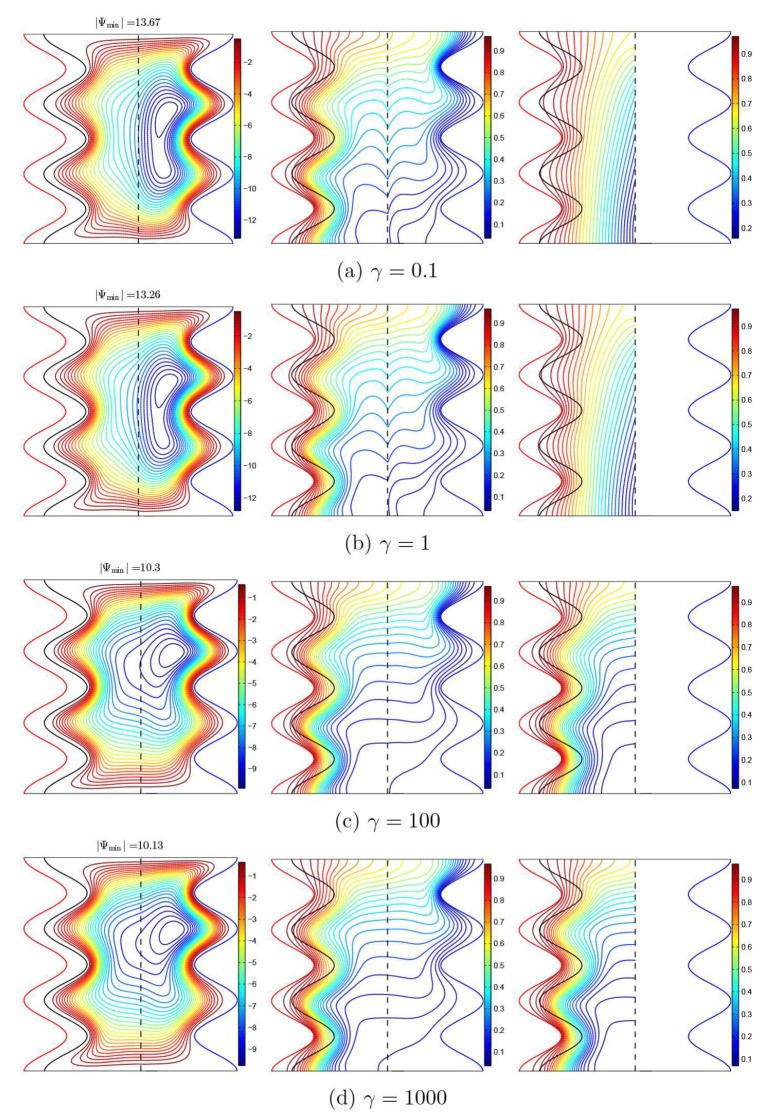
Streamlines (**left**), the isotherms of the nanofluid phase (**middle**), and the isotherms of the solid phase (**right**) with various modified conductivity ratios (γ); $Da=10^{-3}$, ϕ=0.02, A=0.1, and ε=0.5.

The differences in the streamlines patterns and isotherms of the nanofluid and solid phases in the different regions with respect to the thermal conductivity ratio in the porous layer (γ) when $Da=10^{-3}$, ϕ=0.02, A=0.1, and ε=0.5 are depicted in Figure 7. For low values of γ, the core of the flow vortex is found in the nanofluid layer, indicating the nanofluid circulation strength in this region and its weakness in the porous layer region. This is because most of the heat is transmitted through the solid matrix instead of the nanofluid in the porous layer due to the high value of the solid thermal conductivity. The nanofluid-phase and solid-phase isothermal lines are not similar, indicating the existence of the local thermal non-equilibrium case. When raising the value of γ from 0.1 to 1000, the flow vortex expands to cover the porous layer region, and the speed of the circulation strength increases in the porous layer and decreases in the nanofluid layer. It also seems that, as γ increases, the system tends to realize the local thermal equilibrium situation in the porous layer, which is observed by the identicalness between the nanofluid-phase isotherms and those of the solid phase.

**Figure 8:**
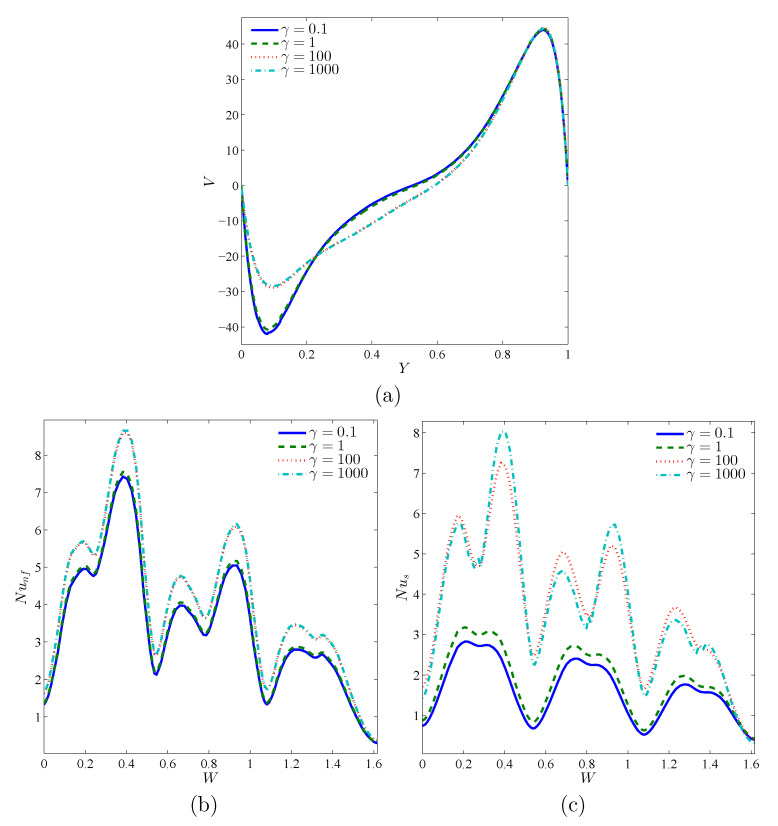
Local velocity (**a**), the local Nusselt number of the nanofluid phase (**b**), and the local Nusselt number of the solid phase (**c**) with the vertical line (*Y*) for X=0.5 for Y=0.5 for different γ values; $Da=10^{-3}$, ϕ=0.02, A=0.1, and ε=0.5.

The local velocity profiles in Figure 8a show that, for low values of γ, the velocity profiles are in conformity because the effect of the solid part is dominant compared to the pore space, which obstructs the buoyancy effects and the flow vortex remains at the center of the cavity. The disparity in the profiles at high γ can be explained by the fact that the flow vortex rises up, which results in a low nanofluid velocity at the lower part of the cavity, near the bottom wall. Figure 8b,c illustrates the impact of the modified thermal conductivity ratio, γ, on both the nanofluid-phase and solid-phase local heat transfer rates. Boosting γ results in an improvement in the Nusselt numbers. For a weak γ, the distribution of $Nu_{s}$ and $Nu_{nf}$ differs, which means that the non-thermal equilibrium effects are significant. In addition, it is to note that the impacts of γ on the Nusselt numbers are more important for the solid phase than for the nanofluid phase. In fact, the attainment of thermal equilibrium between the nanofluid and the solid matrix leads to a greater heat transfer through the entire porous layer.

**Figure 9:**
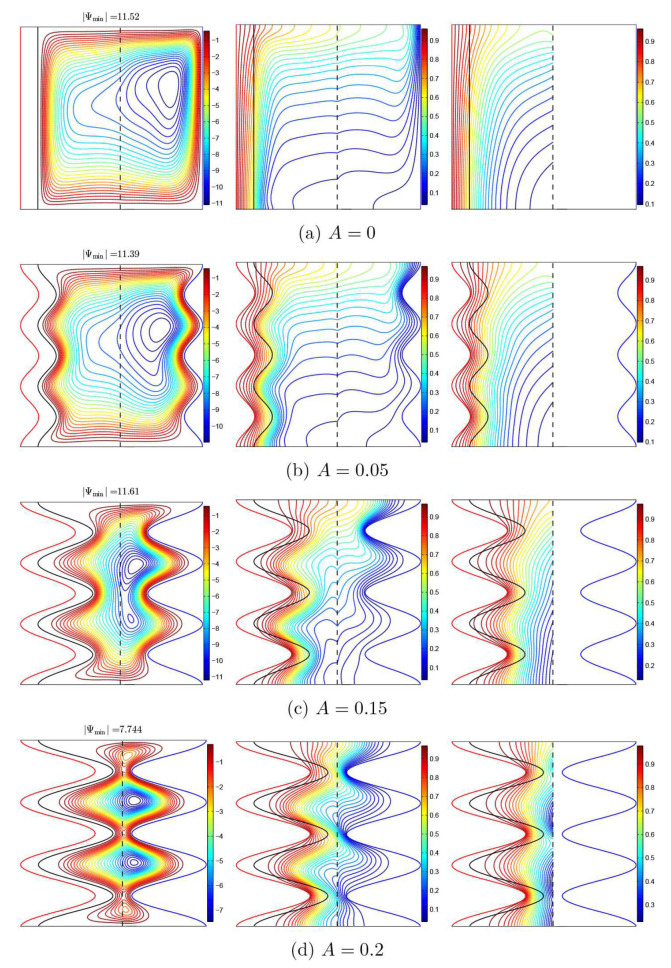
Streamlines (**left**), the isotherms of the nanofluid phase (**middle**), and the isotherms of the solid phase (**right**) with various amplitudes (*A*); $Da=10^{-3}$, ϕ=0.02, γ=10, and ε=0.5.

**Figure 10:**
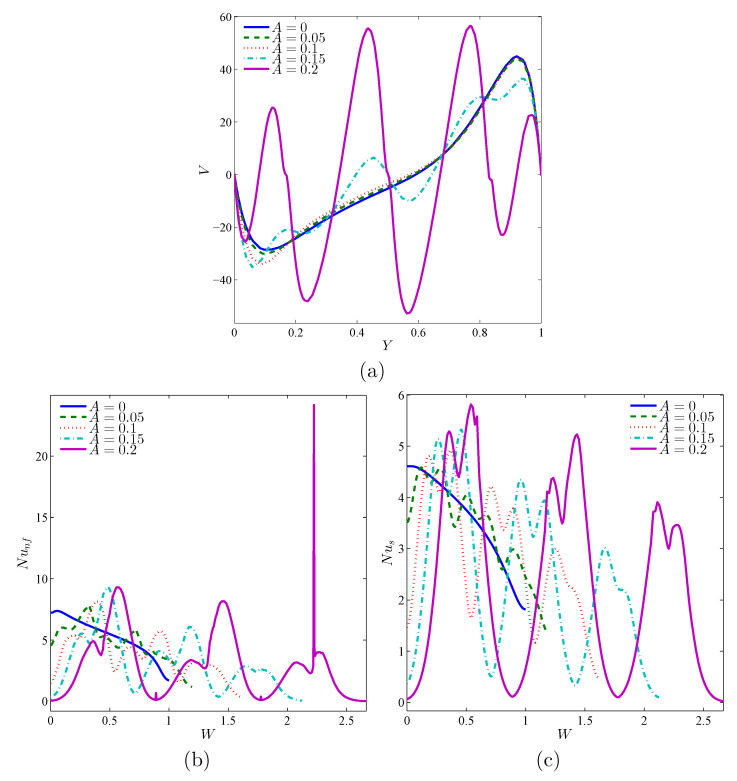
Local velocity (**a**), the local Nusselt number of the nanofluid phase (**b**), and the local Nusselt number of the solid phase (**c**) with the vertical line (*Y*) for X=0.5 for different *N* values; $Da=10^{-3}$, ϕ=0.02, γ=10, and ε=0.5.

Figure 9 analyzes the effect of the magnitude of the undulations of the solid corrugated wall (*A*) on the system’s thermal and dynamic features. Obviously, the larger magnitude undulations, the more conductive the heat transfer tends to be, since the undulations act to impede the nanofluid circulation inside the cavity. The flow configuration switches from one central flow vortex to a multi-core vortex by raising *A*, which influences the distribution of the velocity within the cavity (Figure 10a). Considering the great difference in size and form of the heat exchange surface, the amplitude of the undulations significantly impacts the distribution of the local heat transfer over the hot surface, as seen in the profiles of the Nusselt numbers in Figure 10c,d.

**Figure 11:**
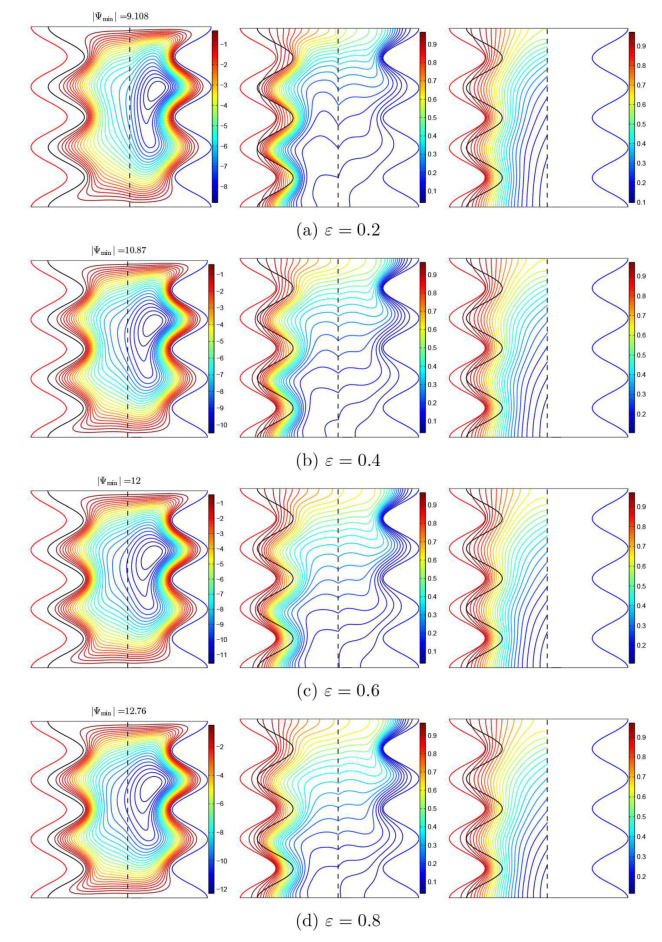
Streamlines (**left**), the isotherms of the nanofluid phase (**middle**), and the isotherms of the solid phase (**right**) with various porosities of the medium (ε); $Da=10^{-3}$, ϕ=0.02, γ=10, and A=0.1.

The varying porosity effects of the porous layer (ε) on the nanofluid and solid phase isotherms and flow patterns in the different regions inside the cavity are demonstrated in Figure 11. As can be seen, an increase in the porosity of the porous layer leads to an increase in the nanofluid circulation within the entire cavity. Indeed, as the porosity increases, the nanofluid movement becomes freer in the cavity, which contributes to a greater heat transfer to the nanofluid layer through the porous layer.

**Figure 12:**
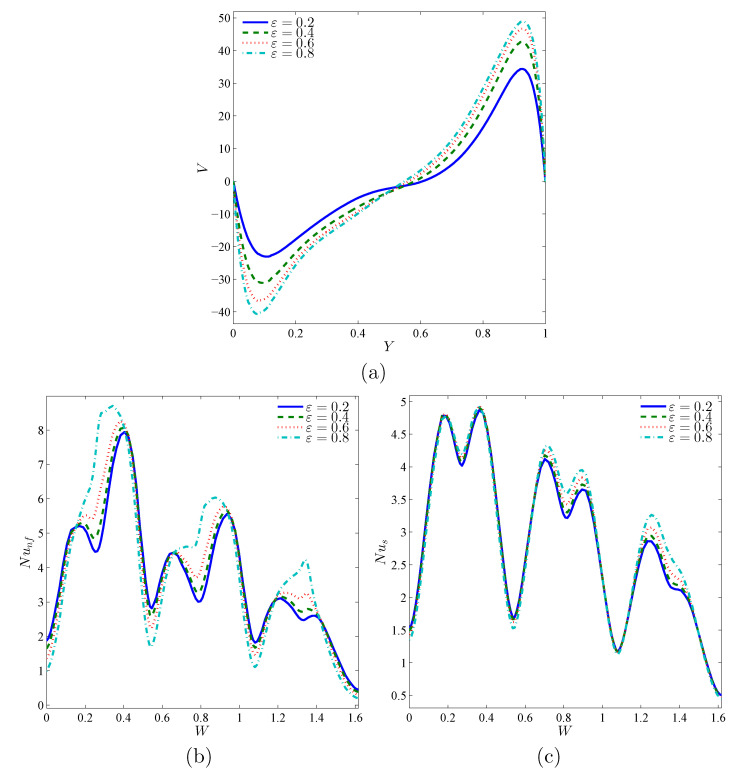
Local velocity (**a**), the local Nusselt number of the nanofluid phase (**b**), and the local Nusselt number of the solid phase (**c**) with the vertical line (*Y*) for X=0.5 for different ε values; $Da=10^{-3}$, ϕ=0.02, γ=10, and A=0.1.

Figure 12a indicates that an improvement in the ε parameter improves the velocity of circulation as well. Figure 12b,c shows that the maximum and minimum of the local heat transfer rates of the nanofluid phase are more extreme with a rise of ε, whereas the rates of the solid-phase heat transfer are not greatly changed by the variations in the porosity magnitude, as the porous media with a large porosity offers more empty spaces to be occupied with the flowing nanofluid.

**Figure 13:**
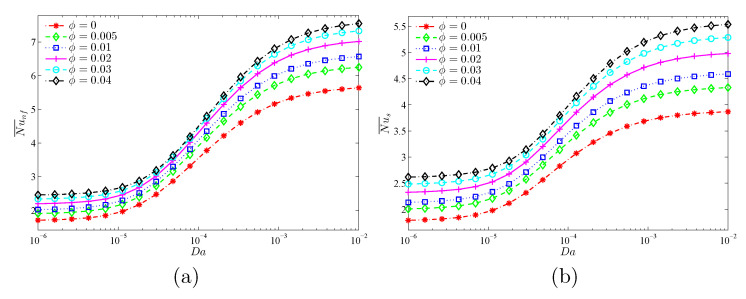
Variations of (**a**) the average Nusselt number of the nanofluid phase and (**b**) the average Nusselt number of the solid phase with Da values for different ϕ values at γ=10, A=0.1, and ε=0.5.

**Figure 14:**
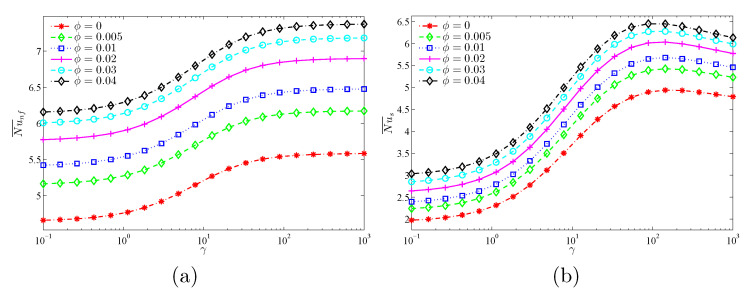
Variations of (**a**) the average Nusselt number of the nanofluid phase and (**b**) the average Nusselt number of the solid phase γ for different ϕ values at $Da=10^{-3}$, N=3, and ε=0.5.

The objective of Figure 13 and Figure 14 is to show the role of the Darcy number (Da) and the modified thermal conductivity ratio (γ) at various nanoparticle concentrations (ϕ) in the average heat transport. It is clear that Da and γ augment the mean Nusselt number of both the nanofluid and the solid phases. The converging values of ${\bar{Nu}}_{nf}$ and ${\bar{Nu}}_{s}$ at lower Da values and higher γ values indicate the local thermal equilibrium situation, as stated earlier. In addition, it is shown that the increasing effect of Da and γ on the mean Nusselt numbers reduces when the Da and γ are higher. Moreover, beyond the value of γ=100, the heat transfer rate decreases with γ because the heat transfer through the solid matrix (${\bar{Nu}}_{s}$) is severely limited due to its low thermal conductivity, unlike ${\bar{Nu}}_{nf}$, which continues to increase with γ due to the improved nanofluid thermal conductivity. It is also evident from the two figures that increasing the nanoparticles’ concentration produces increases in both Nusselt numbers for all considered values of Da and γ.

**Figure 15:**
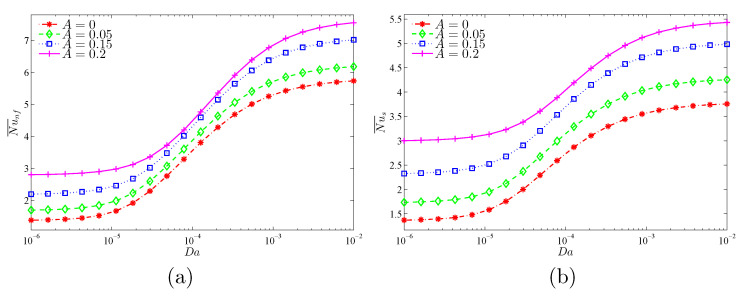
Variations of (**a**) the average Nusselt number of the nanofluid phase and (**b**) the average Nusselt number of the solid phase with Da for different *A* values at ϕ=0.02, γ=10, and ε=0.5.

**Figure 16:**
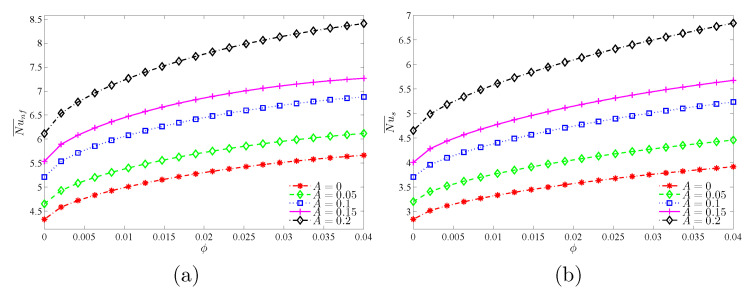
Variations of (**a**) the average Nusselt number of the nanofluid phase and (**b**) the average Nusselt number of the solid phase with ϕ for different *A* values at $Da=10^{-3}$, γ=10, and ε=0.5.

Figure 15 and Figure 16 show that the largest global heat transfer for both the solid and nanofluid phases is found for the highest undulation amplitude (A=0.2) for all tested values of Da and ϕ. This can be attributed to the large heat exchange surface of the wavy wall for high values of *A*. In addition, at a given *A*, the values of the ${\bar{Nu}}_{nf}$ and ${\bar{Nu}}_{s}$ are found to increase with increasing values of Da and ϕ.

**Figure 17:**
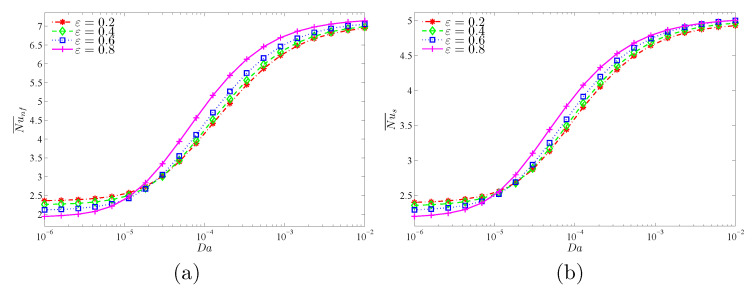
Variations of (**a**) the average Nusselt number of the nanofluid phase and (**b**) the average Nusselt number of the solid phase with Da for different ε values at ϕ=0.02, γ=10, and A=0.1.

Figure 17 aims to examine the role of the porosity (ε) of the porous layer as a function of the Darcy number (Da) in the total heat transfer rates of the nanofluid and solid phases for the case of ε at ϕ=0.02, γ=10, and A=0.1. At lower Darcy numbers ($Da\leq 10^{-5}$), an increase in the porosity of the porous layer weakens the total heat transfer rates (${\bar{Nu}}_{nf}$ and ${\bar{Nu}}_{s}$) owing to the fact that high porosity at low permeability contributes to more heat resistance within the porous medium. The opposite is true for high Darcy numbers.

**Figure 18:**
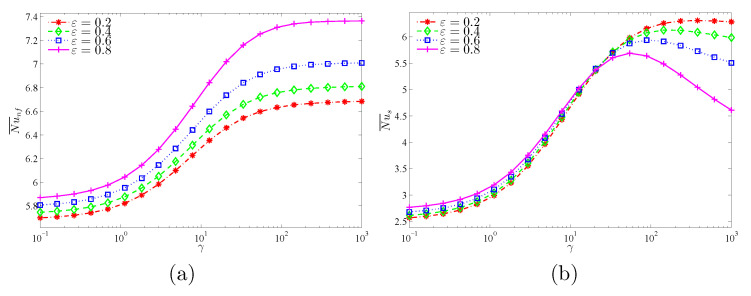
Variations of (**a**) the average Nusselt number of the nanofluid phase and (**b**) the average Nusselt number of the solid phase with γ for different ε values at $Da=10^{-3}$, ϕ=0.02, and A=0.1.

Figure 18 characterizes the variation of ${\bar{Nu}}_{nf}$ and ${\bar{Nu}}_{s}$ with the modified thermal conductivity (γ) for various values of ε. ${\bar{Nu}}_{nf}$ increases with the increment of γ for all values of ε. Meanwhile, there is a limit in the increase of ${\bar{Nu}}_{s}$ with γ after a certain value of ε. This explains that the role of the nanofluid in transferring heat through the porous medium becomes greater than for the solid matrix due to its high thermal conductivity.

## 5. Conclusions

The problem of steady thermal-natural convection in a two-dimensional cavity of corrugated vertical walls consisting of three layers–a conducting solid layer of fixed thickness, a porous medium layer filled with a nanofluid, and a third layer filled with a nanofluid–was numerically studied by employing the finite element method. An alumina nanoparticle-water-based nanofluid was used as a working fluid. The LTNE model was considered for the porous medium. The key conclusions of this analysis are listed below:

1. The local thermal non-equilibrium effects are significant for low values of γ and high values of Da.
2. For low values of Da, the flow is almost entirely confined in the nanofluid layer, and the heat transfer is mainly convective in the nanofluid layer and mainly conductive in the other layers.
3. An increase in Da and ε contributes to an increase in the nanofluid circulation rate in the entire cavity, while an increase in γ causes an increase in the flow circulation in the porous region.
4. An increase in *A* contributes to a decrease in the nanofluid circulation rate in the entire cavity.
5. The best rates of the convective heat transfer through the nanofluid and solid phases are found at high values of *A*, Da, and ϕ for all other constant parameters.
6. The results show an increase in ${\bar{Nu}}_{nf}$ with increasing values of γ, while there is a limit in the increase of ${\bar{Nu}}_{s}$ with γ, especially for high values of ε.
7. ${\bar{Nu}}_{nf}$ and ${\bar{Nu}}_{s}$ decline as ε boosts for low values of Da and enhance for high values of Da.

## Figures and Tables

**Table 1:** Grid testing for $\Psi_{\min}$, ${\bar{Nu}}_{nf}$, and ${\bar{Nu}}_{s}$ at different grid sizes for $Ra=10^{6}$, $Da=10^{-3}$, ϕ=0.02, N=3, γ=10, H=10, ε=0.5, and A=0.1.

Grid Size	Number of Elements	$\Psi_{\min}$	${\bar{\mathrm{Nu}}}_{\mathrm{nf}}$	${\bar{\mathrm{Nu}}}_{s}$
G1	3187	−11.812	6.4267	4.677
G2	3686	−11.691	6.4309	4.6961
G3	4096	−11.676	6.4453	4.7096
G4	4576	−11.603	6.4607	4.7197
G5	5464	−11.591	6.4633	4.7257
**G6**	10,180	−11.528	6.4654	4.7431
G7	21,830	−11.503	6.4655	4.7434

**Table 2:** Thermo-physical characteristics concerning pure liquid (water) and Al${}_{2}$O${}_{3}$ nanoparticles at T=310 K [33].

Physical Properties	Fluid Phase (Water)	Al${}_{2}$O${}_{3}$
$C_{p}\;\left(J/\mathrm{kgK}\right)$	4178	765
$\rho \;(\mathrm{kg}/m^{3})$	993	3970
$k\;({\mathrm{Wm}}^{-1}K^{-1})$	0.628	40
$\beta \times 10^{5}\;\left(1/K\right)$	36.2	0.85
$\mu \times 10^{6}\;\left(\mathrm{kg}/\mathrm{ms}\right)$	695	–
$d_{p}\;\left(\mathrm{nm}\right)$	0.385	33
